# Home Care Nurses at the Heart of the Communication Web: Communication Synchronicity and Effects on the Psychosocial Work Environment

**DOI:** 10.1177/23333936241273145

**Published:** 2024-09-27

**Authors:** Roger Larsson, Gudbjörg Erlingsdóttir, Johanna Persson, Christofer Rydenfält

**Affiliations:** 1 Lund University, Lund, Sweden

**Keywords:** home care, nursing, communication, work environment, interprofessional, teamwork, Sweden

## Abstract

This qualitative study explores how communication practice affects Swedish home care nurses’ psychosocial work environment. Data consisted of interviews and field observations, analyzed from the perspective of Media Synchronicity Theory and the Job-Demand-Control-Support model. Individual home care nurses were found to manage an interorganizational communication web. The results indicated that this web could have a protective function for the nurses. Synchronous communication was found important to control the work situation. Nevertheless, asynchronous communication was enforced when communicating with other health care organizations. This reduced the level of control for the nurses. However, when possible, the nurses also arranged their communication practice to gain control. Thus, local optimization for one group could result in suboptimization for others. We conclude that communication practice should be designed holistically and promote synchronous communication to foster well-functioning interprofessional teamwork and to create a healthy psychosocial work environment for both home care nurses and their collaborators.

## Introduction

In this study we investigate the communication practice of Swedish home care nurses’. Rogers (1986, p. 199) defines communication as “a process in which participants create and share information with one another in order to reach a mutual understanding.” Meaning is conveyed verbally and nonverbally by sending and interpreting information to achieve shared understanding among individuals within specific contexts (Daft & Lengel, 1986; Miranda & Saunders, 2003; Rigotti & Rocci, 2006; Rogers, 1986).

Communication is an important part of nursing practice (Kieft et al., 2014; Kourkouta & Papathanasiou, 2014; Manojlovich & DeCicco, 2007). However, previous research has indicated that it can be a source of inefficiency and disturbance (Persson et al., 2023; Tjia et al., 2009; Vermeir et al., 2015). The importance of communication for teamwork and collaboration in health care is well-documented. Among other things, communication is associated with increased patient safety and care quality (Härgestam et al., 2013; Hewitt et al., 2015; Larsson et al., 2022; Lingard et al., 2006; Rydenfält et al., 2017; Suter et al., 2009). However, gaps and breakdowns in health care communication remain common. The ever-advancing specialization of health care has increased the gaps between areas of expertise (Cook et al., 2000). At the same time, increased care complexity that comes with more advanced care procedures makes it more important than ever for professionals across different areas of expertise to work together (Reeves et al., 2010). This along with the aging world population and the related comorbidities that come with age, present challenges for today’s health care systems (United Nations Department of Economic and Social Affairs, Population Division, 2020). Due to the aging world population, the need for health care to be delivered at home is expected to grow worldwide and enable patients to live at home for longer (Genet et al., 2012; Nordic Council of Ministers, 2014; United Nations Department of Economic and Social Affairs, Population Division, 2020). Additionally, digitalization is seen as necessary to enable treatment and care at home (De Raeve et al., 2017; Kamp et al., 2019). The effects of more interdependent and digitalized health care, and the expected need to move health care outside of traditional settings and institutions, makes the context of care and work more diverse. It is thus of considerable interest to study communication practices among professional groups in and across different health care contexts.

The care responsibility of the Swedish public health care system is divided. Hospitals and community health centers are run on the regional level. Municipalities are responsible for health and social care related to prevention work, care for the elderly and other citizens in need (Andersson & Karlberg, 2000; Nordic Council of Ministers, 2014). Swedish municipal organizations cover a variety of specialized subfunctions, one of which is municipal care. Municipal care in Sweden can be contextually delivered in a variety of locations. This can range from ordinary homes to specialized living arrangements such as nursing homes, homes for the aged, or housings for the elderly. This study has been conducted on the working conditions among nurses that provide care for patients living in their own homes, that is, municipal home care. In municipal home care, nurses reside as a medical profession governed by the Swedish Health and Medical Care Act. Physicians only work within the regions, because municipalities are not allowed by law to hire them for patient-related work. Thus, in Sweden, the ability to deliver municipal home care is a combined effort from professionals of different disciplines and organizations that work together to deliver health care services (often long-term) to citizens in local communities. In practice, home care nurses often deliver care on their own in the patient’s home without direct access to nursing colleagues or physicians.

Registered and community health nurses in municipalities (from here on referred to as “home care nurses”) manage much of the communication between professionals involved in the care of patients at home. This involves communication through a multitude of media, both within and across organizational boundaries (Persson et al., 2023). Home care nursing has previously been described as a networking activity where nets of supporting professions are created around each patient (Bjornsdottir, 2018; De Groot et al., 2018). This networking role is also something that home care nurses consider makes their work attractive (De Groot et al., 2018). Thus, the networking and communication practice of home care nurses is central to their work. However, little is known about how the day-to-day communication of home care nurses is managed and how characteristics of the communication affect their psychosocial work environment. Here, the psychosocial work environment refers to all aspects of the individual’s work situation that affects them on a psychological or social level. Since a healthy work environment is associated with improved care quality and job satisfaction (Ma et al., 2015; White et al., 2020), and communication is an integral part of nursing practice and teamwork, it is of considerable interest to explore how the communication practice can affect the work environment.

### Aim

This study investigated home care nurses’ communication practice. We were particularly interested in the characteristics of this practice and its effects on the psychosocial work environment. We approached this issue with a combined theoretical framework consisting of the *Media Synchronicity Theory* (MST) and the *Job-Demand-Control-Support* model (JDCS) (Dennis & Valacich, 1999; Dennis et al., 2008; Johnson & Hall, 1988; Karasek, 1979; Karasek & Theorell, 1990). Specifically, the aim of this study was:

1) To explore the communication practice of home care nurses.
2) To investigate how the type of communication media and its synchronicity influence the nurses’ communication practice.
3) To investigate how home care nurses’ psychosocial work environment was affected by the media choice.

## Theoretical Framework

The following two sections describe the two theoretical frameworks that we utilized: MST and JDCS. The MST was chosen as it informs the analysis of how communication practice is influenced by the degree of media synchronicity. The JDCS model was chosen as it sheds light on the workers situation and how their psychosocial work environment affects them.

### Media Synchronicity Theory (MST)

The MST addresses “. . . the ability of media to support synchronicity, a shared pattern of coordinated behavior among individuals as they work together” (Dennis et al., 2008, p. 575). Some media can be used synchronously, which means that the parties are communicating at the same time. Other media are asynchronous, which means that the parties are not communicating at the same time. And still other media are either synchronous or asynchronous depending on how they are used. Dennis et al. (2008, p. 581) define a medium’s synchronicity as “the extent to which the capabilities of a communication medium enable individuals to achieve synchronicity.”

The MST highlights how media synchronicity can support two primary interpersonal communication processes: *conveyance* of information and *convergence* of meaning (Miranda & Saunders, 2003). Dennis et al. (2008, p. 580) define conveyance as “the transmission of a diversity of new information . . . to enable the receiver to *create* and *revise* a mental model of the situation.” They define convergence as “the discussion of preprocessed information about each individual’s *interpretation* of a situation, not the raw information itself.” Conveyance concerns the transfer and subsequent retrospective analysis of raw information. Convergence is about creating a compatible understanding of high-level abstractions, of what to communicate, and of what needs to be done. In other words, to establish and/or adjust shared mental models. Conveyance processes are better supported by media that offer low synchronicity. Convergence processes are better supported by media that offer higher synchronicity. We used MST to investigate how the dynamics and characteristics of communication media can provide home care nurses with conditions to convey or converge on information in their daily work.

### Job-Demand-Control-Support Model (JDCS)

**Figure 1.**
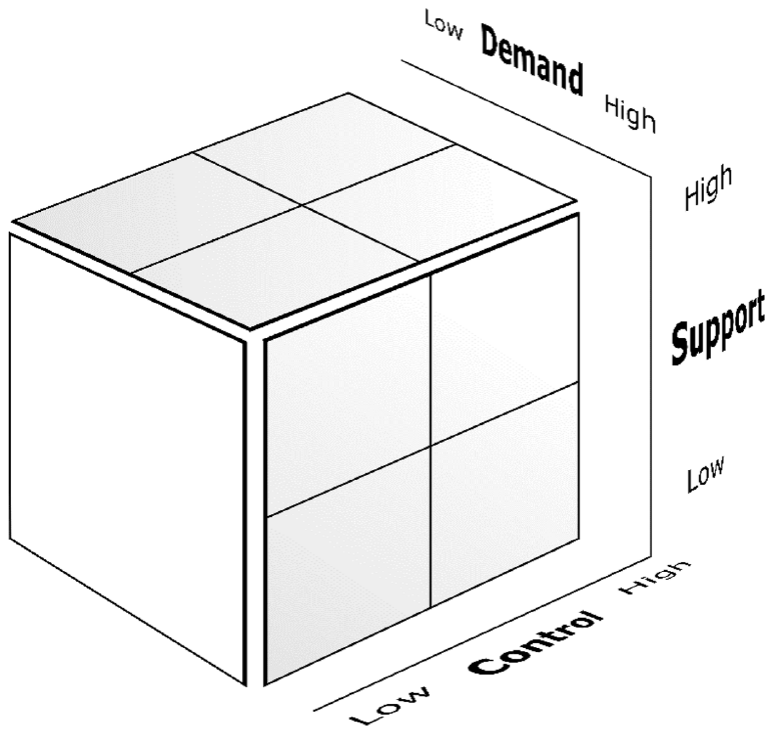
The Job-Demand-Control-Support (JDCS) model after Karasek and Theorell (1990).

The JDCS model concerns the relationship between working conditions and stress (Johnson & Hall, 1988; Karasek & Theorell, 1990). The JDCS model is one of the most popular and well-researched models that describe job strain. According to the model, psychological strain (stress) is a result of multiple factors present in the work environment: demand, control (decision latitude), and support. These three factors can be arranged in a three-dimensional property space (see Figure 1). A work environment is considered to be healthy when people experience high enough levels of control and support to meet the demands presented by their work. As demands increase, so does the need for control. The support factor acts as a buffer when control decreases. Work becomes unhealthy, even dangerous, when workers are exposed to demand levels that they experience as being too high in combination with low levels of control and support. However, work demand levels that are too low are not considered to be beneficial either because this has a negative effect on work motivation.

We used JDCS to investigate how demands, control, and support for home care nurses were facilitated through the communication practice used in their work.

## Methods

### Research Design

The research design of this qualitative study has much in common with a qualitative descriptive approach (Bradshaw et al., 2017). This approach fits the aim well as the study takes its departure in a description and exploration of the home care nurses communication practice. The design also was informed by ethnographic methods (Czarniawska, 2007; Emerson et al., 2011). Specifically, it resembles what sometimes is described as a focused ethnography, that is, an ethnography focused on specific aspects of the field rather than the field as a whole (Higginbottom et al., 2013).

Semi-structured interviews and field observations in the form of shadowing were used to explore the communication practice of Swedish home care nurses (Czarniawska, 2007; Kvale & Brinkmann, 2009). The study was conducted as part of a research project focused on interprofessional teamwork in municipal home care. The semi-structured interviews provided knowledge about the nurses’ thoughts and experiences of communication. The field observations, inspired by ethnographic methods, provided accounts of how communication took place in practice (Czarniawska, 2007; Emerson et al., 2011).

### Sample and Data Collection

The data were collected from January to May 2020. The data set consisted of 22 semi-structured interviews and 10 field observations. All participants were employed in Swedish municipal home care.

Twenty interviews were conducted with one nurse at a time. Two were conducted with two nurses at the same time. In total, 24 nurses (3 men, 21 women) were interviewed by the first, second, and fourth authors. The interviews encompassed participants from four Swedish municipal home care organizations. They were recruited with the help of each home care organization’s unit manager. The interviews lasted between 27 min and 1 hr 19 min (total = 16 hr, mean = 43.6 min). The interviews were designed around the following topics: *Mobility in work*, *Organizational change to mobile teams*, *Team composition*, *Change of work*, *Use of digital tools and eHealth systems*. The semi-structured interviews were guided by a predefined interview guide consisting of questions about these topics. Some of the questions addressed communication directly, for example:

- *How do you normally communicate with your colleagues when you are out in the field? Give some examples of what may need to be communicated.*
- *How often do you usually need to have contact with a general practitioner?*
- *Do you contact a general practitioner every time you would like medical support?*
- *How does it work when you contact or come into contact with the mobile team?*

Communication could also be addressed indirectly as part of the conversation regarding other questions. The interviews were audio recorded. The interviews were conducted in person up to the beginning of March 2020. After that, they were replaced by telephone interviews. This transition was necessary to handle the COVID-19 outbreak. While the participating municipalities were mildly affected by the first wave of the pandemic, visits were strictly prohibited starting in March 2020.

The 10 field observations were conducted in two municipalities before being canceled due to visiting restrictions. The first author performed all the field observations. The documentation of the observations consisted of handwritten field notes taken while observing the nurses’ work. In total, 10 nurses (2 men, 8 women) were observed. Eight out of the 10 observations covered the nurses’ entire shift. One observation was split into two due to a switch of the home care nurse being shadowed. For the observations, the nurses were recruited by the first author during the interviews or through recommendation by the unit manager. The observations lasted between 3 hr 10 min and 9 hr 40 min (total = 79 hr 55 min, mean = 7 hr 59 min). The observations were guided by a list of predefined topics, including: *Communication with colleagues, Support from colleagues and others*, *Obstacles at work*, *Use of digital tools and eHealth systems*, and *The importance of the teams for the work.* Ethical considerations were continuously made of what and how to document, especially while following the nurses during home visits. The field notes were structured chronologically, i.e. it was noted when things happened during the shift.

### Data Analysis

All the interviews were transcribed. The handwritten field notes were converted into digital form. There were two phases in the analysis process: a data driven phase consisting of qualitative content analysis, and a theoretically driven phase where the output from the first phase was scrutinized through the lenses of MST and JDCS. A conventional approach to qualitative content analysis was used to analyze the data (Elo & Kyngnäs, 2008; Hsieh & Shannon, 2005). Inductive qualitative content analysis was chosen due to the study’s explorative and descriptive nature in the data driven phase (Elo & Kyngnäs, 2008; Vaismoradi et al., 2013). QSR NVivo R1© was used for the qualitative content analysis.

The data driven qualitative content analysis phase was comprised of three steps, with iterative elements in between, allowing the emergent codes to be revised during the process.

(1) The first author read the interview transcripts and observation field notes. This represents the preparation phase according to Elo and Kyngnäs (2008).
(2) Eight interviews and field notes from two observations were initially openly coded by the first author. No a priori coding scheme was used. The result was a broad set of codes that were sorted into categories by the first author. This represents the organizing phase according to Elo and Kyngnäs (2008).
(3) All authors discussed the open coding and categories and decided to further develop the *Communication* category because of its dominance. This included:
    a. The open coding of the entire data set (22 interviews and 10 field observations) from the perspective of communication. Here, the unit of analysis, was refined, i.e. we decided to focus solely on the communication practice of nurses. This represents a new iteration of the organizing phase (Elo & Kyngnäs, 2008).
    b. Refining the category and breaking it down into subcategories of *Recipients of communication*, *Media used for communication*, and *Type of communication enacted through media*. Detailed annotations were made during this process. This represents the grouping and categorization parts of the organizing phase (Elo & Kyngnäs, 2008).

The theoretically driven analysis phase was comprised of two steps:

(1) Assessment of the communication synchronicity of key media used from the perspective of MST.
(2) Analysis from the perspective of JDCS of the possible implications for the nurses’ psychosocial work environment based on the use of different media.

### Ethical Considerations

Before data collection started, interview and field observation participants received information about the study and were told that they could withdraw at any time. Each participant also signed an informed consent form. This study received ethical approval by the Swedish Ethics Review Authority (DNR 2019-04653).

## Results

The home care nurses were found to partake in a complex communication practice which was integral to their daily work. This involved communication with a vast array of professional groups in different settings and through different media. In order to proactively address problems early, each nurse managed interorganizational communication pathways around their patients. We have conceptualized this as a “communication web”: comprised of nodes (people) and connections (media). Three distinctive types of communication, represented by thirteen media, were used by the home care nurses: oral, written, and visual communication. An overview of the identified media is presented in Table 1.

**Table 1.** An Overview of Prevalent Media Used by Home Care Nurses, What Type of Communication They Represent, and the Associated Level of Synchronicity in Accordance With Dennis et al. (2008).

Media	Type of communication	Synchronicity
Face-to-face meetings	Oral	High
Phone calls		Medium
Handwritten notes	Written	Low
Forms/physical documents		Low
Texting		Low
Faxing		Low
EHR documentation		Low
E-mail		Low
EHR messaging		Low
Digital medical signing		Low
Communication portals		Low
Picture messaging	Visual	Low
Photographs in EHR documentation		Low

**Figure 2.**
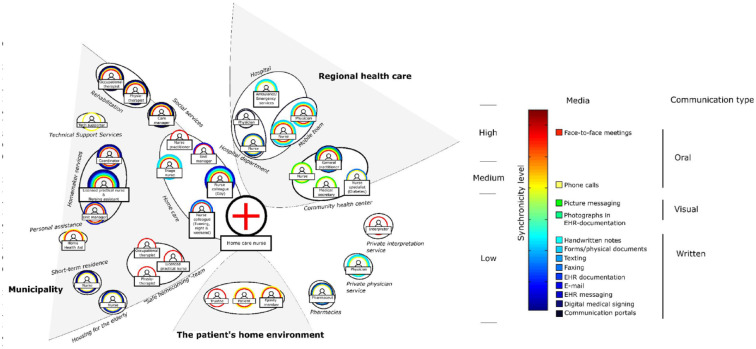
The visualized “communication web” of nurses in municipal home care. The color of the rings surrounding each professional group represents the media used for communication. More rings indicate a wider range of media used. The color indicates the level of synchronicity.

A visualization of the communication web is found in Figure 2. The home care nurses acted as a link between professional groups in the web, otherwise not in direct contact with each other, for example general practitioners at community health centers and licensed practical nurses in homemaker services. Furthermore, the home care nurses were often found to be the driving and coordinating force of communication.

The analysis of the three types of communication and their potential effects on the work environment will be presented in the following sections.

### Media Used in Three Types of Communication and the Potential Effects on the Work Environment

Tables 2 to 4 present examples of typical interactions involving oral, written, and visual communication between nurses and four common actors within the nurses’ communication web identified in the data: *homemaker services*, *home care nurse colleagues, community health centers* and *hospitals*. These quotes are positioned in the middle of the tables. The tables are read from left to right. To the left, the quotes and media are analyzed from the perspective of the MST framework in terms of synchronicity in accordance with Dennis et al. (2008) (low, medium, high). To the right, we identify how and why the psychosocial work environment of home care nurses was affected by the communication practice from the perspective of the JDCS model (Karasek & Theorell, 1990).

**Table 2.** Examples of Communication Through Oral Media and Their Effects on the Psychosocial Work Environment.

Media and synchronicity levels. MST perspective.	Examples of interactions from observation field notes and interviews	How communication through oral media facilitates demands, control, and support at work. JDCS perspective.
**Face-to-face meetings** High	**Homemaker services** **Routine morning meeting with the homemaker service group** *“[The home care nurse] begins the morning meeting with . . . her homemaker service group where . . . [today’s] events and important information about patients [are reviewed]. The home care nurse writes down notes in her calendar of what she needs to pass on to others, such as the [occupational] and physiotherapists, or to [the patient’s] relatives.”—Observation field note* **Spontaneous meeting with a licensed practical nurse during home visit** *“[A licensed practical nurse (LPN)] from the morning [meeting] . . . arrives [inside the patient’s home] to drop off [a] prepackaged role of medication . . . She informs the home care nurse that today’s schedule is tough: “I don’t know how they thought about the round today.” The LPN asks the home care nurse if she . . . [could]. . . give the patient the medication instead, which [the nurse agrees to do to help the LPN’s stressful situation].”—Observation field note*	*The nurse and homemaker service group mutually* ***demand*** *information from each other. They collectively provide mutual* ***instrumental support*** *and thus take* ***control*** *over their future tasks and codependent work situation.* *The home care nurse perceives a* ***demand*** *to give* ***emotional*** *and* ***instrumental support***. *In doing so, she acts proactively to lower the risk for negative consequences and loss of* ***control*** *to occur due to the LPN’s strained work situation.*
	**Home care nurse colleagues** **Spontaneous meeting with a home care nurse colleague between home visits** *“[Cycling] on her way to a home visit [where the general practitioner (GP), patient and patient’s daughter will be present], the home care nurse meets . . . [one of her office] colleagues driving a car. The colleague gets . . . out of the car after parking it on the side of the road . . . The two nurses start talking about how [work] is going and how things are with [their] patients. N.N. [informs the colleague that she needs to perform an emergency bandage change after receiving a phone call that one of her] patient’s sores [has] bled through the bandages. [The colleague offers to take over that task for N.N., so she can focus on the upcoming meeting. The nurses negotiate back and forth] . . . The colleague ends up taking on the bandage change task. . . Both nurses agree on this arrangement and . . . go their separate ways.”—Observation field note*	*The nurse receives* ***instrumental support*** *from her colleague. By redistributing tasks among themselves, the workload is equalized. This lower* ***demands*** *on the nurse while simultaneously restoring her* ***control*** *over her work situation. This also increases the local nursing group’s* ***control*** *over their shared work situation.*
	**Community Health Center** **Preparations for routine medical rounds with a general practitioner** *“I usually keep [errands about enrolling patients into the mobile team and updating coordinated care plans] . . . until the times [when I and the general practitioner (GP)] meet at the community health center once a week. That way there won’t be . . . a lot of faxing and phone calls [back and forth]. . . [T]hen I usually write down [and send, e.g.,] the prepared care plans, follow-ups, and the like [to the GP] so that you take everything together. [Otherwise] it’s likely [things] get lost, you don’t really get feedback that way either.”—Interview* **Routine medical round with a home care nurse colleague and two general practitioners at community health center** *“Two general practitioners (GPs) arrive . . . in the meeting room and sit down together with [one home care nurse each] . . . [In pairs, the participants] go through patients. One of the GPs [is stressed due to an ongoing patient visit] and leaves after 5 min. The others [continue] . . . The second GP leaves 2 min later to check up on something. Nine minutes pass, and the second GP returns to talk with the nurses for another 3 min before he finally leaves] . . .”—Observation field note*	***Demands*** *are put on the nurse to provide/present thorough documentation to the GP before the meeting. Structure is created to enable* ***control*** *over the outcome when the nurse requests* ***instrumental support*** *related to her work.* *Two home care nurses seek* ***instrumental support*** *from their respective GPs. The nurses have little* ***control*** *over the duration of the meeting and its outcome since the GPs leave on their terms. This increases the* ***demands*** *for the nurses to be well prepared before the meeting and to the point with what they want.*
**Phone calls** Medium *In practice low with community health centers	**Homemaker Services** **About making phone calls to homemaker services during a home visit** *“. . . And homemaker services if I have any questions [when I’m with] the . . . patient. Perhaps they’ve already been there [earlier], and I . . . don’t understand why they haven’t done [a specific task], or I want to ask [about] something. Then I usually call homemaker services . . .”—Interview*	*When* ***demands*** *make it necessary for the nurse to get more information, the nurse requests* ***instrumental support*** *from the homemaker service group to gain the information. Thus, the nurse takes the initiative and strives to increase her* ***control*** *over the task.*
	***Home care nurse colleagues*** **Phone calls with colleagues at or between home visits** “It could be that I forgot some materials [at the office]. Then I call my colleagues who . . . I know are nearby and see if they have brought it along . . . Or it could be a patient I’m stuck with, and I’m running out of time for my other tasks . . . If I must be some place at a particular time . . . [Then my colleagues can help me out] . . . Or it could be that I’m simply stuck and need help with a patient [because I cannot handle the situation myself].”—Interview	*The nurse seeks* ***instrumental support*** *from her colleagues when* ***demands*** *become too great for her to handle by herself. In doing so, the nurse regains* ***control*** *over her task and work situation with the help from her colleagues.*
	***Community Health Center*** **Phone call through a triage nurse for allowance to contact physicians** “[When I’m to contact the general practitioner (GP)] according to the routine, I must talk to the triage nurse (TN) at the community health center first. If . . . the patient’s [GP] is available for a phone call . . ., then I can call [him] directly myself, but it rarely matches timewise . . . [If it feels] . . . a bit more urgent, then I have to call either the regional mobile care team or [a private physician firm] for a home visit. Then I must talk to the TN at the community health center first before I can call them.”—Interview **Phone call with general practitioner through a triage nurse** “[If] I’ve been at a home visit where I feel [a patient is] accumulating [fluid]. . . [Then you might want to] . . . have an assessment [for] heart failure [or] something like that. It’s not urgent, so I’ll take care of it when I’m back [at the office]. Then I call the general practitioner (GP) or . . . we have a VIP number that we call. [Then] I have to talk to the triage nurse (TN) [at the community health center] and ask her to ask the GP to call me.”—Interview	*Home care nurses experience needs to seek* ***instrumental support*** *related to their tasks, but they must wait for GPs to get in touch or ask for permission to contact other health care instances. Communication with the TN is synchronous but asynchronous with e.g., GPs. The home care nurses are* ***demanded*** *to ask for permission, wait or even follow up communication if needed. This results in a lack of* ***control*** *as to what they are allowed to do and when they will receive an answer.* *The communication synchronicity of phone calls with GPs do not follow MST. The home care nurse-GP interaction is asynchronous, i.e. of low synchronicity. Message passing takes place rather than a direct phone call. The act of communication is pushed to a lower synchronicity level.

**Table 3.** Examples of Communication Through Written Media and Their Effects on the Psychosocial Work Environment.

Media and synchronicity levels. MST perspective.	Examples of interactions from observation field notes and interviews	How communication through written media facilitates demands, control, and support at work. JDCS perspective.
***Handwritten notes*** *Low*	**Homemaker Services** **Writing a note to the homemaker service group** *“[The home care nurse] drops off the bag of materials at [the office of] her homemaker service group. The materials include dressing material, bandages [and] anesthetic gel for a catheter. The nurse writes . . . a note, informing the homemaker service personnel [which patient the bag of materials is for] and puts [the note] on top of the bag.”—Observation field note*	*The home care nurse experiences a* ***demand*** *to provide complementary information to where the materials are intended to be delivered. She provides* ***instrumental support*** *by writing instructions. By leaving the note on top of the bag of materials, the nurse gains* ***control*** *by delegating and instructing where the materials are to be delivered.*
	**Home care nurse colleagues** **Reading written instructions during a routine morning meeting with a colleague’s homemaker service group** *“[The home care nurse] goes . . . to [her colleagues] homemaker service group. [With her] she has two calendars, her own and . . . her colleague’s. [The colleague is off duty, so she covers for her today.] The nurse starts the meeting and goes by what the notes [in her colleague’s calendar] say are important to remember for the day.”* *– Observation field note* **Requesting written instructions in the colleague’s calendar** *“[The home care nurse] converses with her colleague [about] the plans for tomorrow. The colleague will be off work [and the home care nurse will cover for her] . . . so they go through [the planned tasks together], one at a time. The colleague has written some instructions in her calendar . . . for the home care nurse to follow. After reading the instructions, and as they are talking about the tasks, the home care nurse asks her colleague [to] write down extra instructions.”—Observation field note*	*The situation* ***demands*** *the home care nurse to brief her colleague’s homemaker service group.* ***Instrumental support*** *is received through well-written notes in the colleague’s calendar. This increases the nurse’s* ***control*** *as she successfully can lead the briefing.* *The nurse requests elaborate* ***instrumental support*** *through written notes from her colleague. This enables increased* ***control*** *for the nurse over tasks for the next day, giving her quick access to more information related to the tasks. Thus*, ***demands*** *for the nurse to look up information decreases.*
***Texting (SMS)*** *Low*	**Homemaker services** **About leaving text messages (SMS) when unavailable** *“Many times, [homemaker services text] me if I’m busy and don’t answer [the phone]. Like if something needs to be ordered or [if] they want [something].”—Interview* **About avoiding texting in practice (SMS)** *“[T]here may arrive . . . texts from the licensed practical nurses (LPNs) in homemaker services. But we try not to have that form of communication . . . It’s easy that you forget to look, you hear the ping, but you are too busy when you hear it . . . and then it’s gone from your mind because it doesn’t ping several times to remind you . . . Then it’s better to call [if you] want something and it’s urgent. If it can wait, then wait until we come back to the office, because you are distracted from your patient every time you are interrupted . . . And if it’s about confidential things, then you must go outside and talk about it, and so on. So, if it’s not urgent we prefer to take things when we get back to the office. Either [that] they leave notes, call us, . . . or just come upstairs and talk to us face-to-face.”* *– Interview*	*The home care nurse provides an option for mutual* ***instrumental support*** *if not available. This increases the ability to address tasks fast and gives the nurse more* ***control*** *over when to address and handle tasks. It also decreases* ***demands*** *for availability over the phone right away.* *This home care nurse wants to limit the number of possible media for communication, that is, to lower her availability. By so doing, the nurse increases her* ***control*** *over where and when to anticipate communication. At the same time, she lowers the risk for communication breakdowns. This also decreases the nurses’* ***demands*** *to check different media for the same information.*
***Faxing*** *Low*	**Community Health Center** **Having to wait for responses from general practitioners through fax, nr 1** *“If [the community health center’s availability] is good, we can get in touch with a general practitioner (GP) and talk to [him] over the phone on the same day, or [even better] at the same time, [that we] call. Otherwise . . . you may have to wait . . . [You can] fax, and then it may take several days before you get an answer.”—Interview* **Having to wait for responses from general practitioners through fax, nr 2** *“We try to contact [the general practitioners (GPs)], but it’s . . . by fax . . . then some get sick, others aren’t there that day, and the [fax forms] start circulating around to other general practitioners.”—Interview*	*Home care nurses are* ***demanded*** *to communicate via fax with community health centers if tasks are non-urgent. The nurses seek* ***instrumental support*** *from GPs frequently at work. Yet they have little to no* ***control*** *of knowing when an answer will return. In addition, the nurses have no way of knowing if someone handles their request for support or not.*
	**Hospital** **Short notice notification via fax of patient discharge** *“A fax [has arrived from the hospital] . . . during the afternoon. After reading it, the home care nurse hands it over to . . . her colleague. The fax is about . . . [a] patient who is coming home tomorrow [on short notice]. [The notice has taken home care and homemaker services completely off guard. Earlier today, another patient had come home from inpatient care too.] “We don’t want any more.” . . . the colleague exclaims. . . . [The feeling is that] the hospital does not consider the municipality’s [limited preconditions to receive patients with short notice].”—Observation field note*	*The home care nurses experience no* ***control*** *over the situation, while still being* ***demanded*** *to handle the situation as best they can. The nurses have no possibility to seek* ***instrumental support*** *from the hospital, thus they seek* ***emotional support*** *from each other.*
***EHR documentation*** *Low*	**Home care nurse colleagues** **Reading documentation as a means of communication** *“Every morning . . . I try to read [the documentation for] what has happened since I previously left my shift. [T]his can be over a weekend, or from yesterday afternoon.”—Interview*	*Work* ***demands*** *the home care nurse to be updated about the care situation of her patients. By reading documentation on past events*, ***instrumental support*** *is gained, as well as* ***control*** *over coming tasks.*
***EHR-messaging*** *Low*	**Homemaker services** **Initiation of task for coordinator in homemaker services** *“The home care nurse writes a message through the EHR system [to the homemaker service coordinator] about adding a new entry in the digital medical signing system for redressing a wound on the patient’s nose.”* *– Observation field note*	*The home care nurses do not have access to the homemaker services coordination system. Thus, they are* ***demanded*** *to contact the homemaker service coordinator through the EHR system. This nurse reaches for* ***control*** *over the task by requesting* ***instrumental support*** *from the coordinator.*
***Communication portals*** *Low*	**Community Health Center** **Testimony during a scheduled meeting among home care nurse colleagues on complications of messaging general practitioners** *“Two home care nurses address [the problem to the group] that . . . general practitioners (GPs) do not . . . update/approve new coordinated care plans in the communication portal. One of the nurses shares that she has reminded [one] GP [to] . . . approve [one patient’s] coordinated care plan three times, but . . . nothing has happened. She continues by saying that she has documented this in the local EHR system . . . [and that it] has all resulted in that the patient in question has now been hospitalized.”—Observation field note* **Absent communication from general practitioner about prescription changes** *[The home care nurse goes] into the drug prescription service system to look at [a] patient’s medication list. The GP has made changes to the medication list without notifying home care . . .—Observation field note*	*The home care nurses get little* ***instrumental support*** *from GPs with the completion of the agreed coordinated care plans. Hence, the nurses lose* ***control*** *over their work situation and the* ***demands*** *to follow-up communication and handle consequences increase.* ***Instrumental support*** *from the GP is absent when prescriptions are changed in the drug prescription service system. Home care nurses have little* ***control*** *over if changes have been made, thus they are* ***demanded*** *to continuously check the system and handle changes on short notice.*

**Table 4.** Examples of Communication Through Visual Media and Their Effects on the Psychosocial Work Environment.

Media and synchronicity levels. MST perspective.	Examples of interactions from observation field notes and interviews	How communication through visual media facilitates demands, control, and support at work. JDCS perspective.
***Picture messaging (MMS)*** *Low*	**Homemaker services** **Receiving photographs from licensed practical nurses** *“It’s mostly the licensed practical nurses (LPNs) in homemaker services who report to us . . . when they discover something, they report it [through photos] so you can tell them directly what to do. [It’s a big help] to them . . . when they’re standing there. Additionally, we don’t have to drive out to have a look. Instead, we may answer straight away, ‘No, but that’s how it looked yesterday too, so there’s no danger’. So sure, it can save us many unplanned home visits.”—Interview*	*Home care nurses provide* ***instrumental support*** *to homemaker services based on photographs.* ***Demands*** *for the nurse to travel and do unplanned home visits decrease.* ***Control*** *is retained over the nurses’ planned work situation.*
	**Community Health Center** **Requesting opinion from general practitioner** *“At times we can tell the general practitioner (GP) that . . . ‘Well, we have a photo . . . [Can] you look at . . . this rash? What do you think?’ Otherwise, you stand there and have to explain what the rash looks like and such . . .”* *– Interview* ***Limited infrastructure to communicate with general practitioner*** *“So I take a photo of the wound . . . which I want to give to the triage nurse (TN) [at the community health center], who in turn will give it to the general practitioner (GP) to look at. But where should I send it? It ends up being sent [to] their private phone or something like that. The technology doesn’t sync very well.”—Interview*	*The home care nurse asks for* ***instrumental support*** *from a GP. The* ***demand*** *to convey information orally decreases. This helps the nurse to gain* ***control*** *over her task.* *The home care nurse seeks* ***control*** *over her task, but experiences limitations to receive desirable* ***instrumental support***. ***Demands*** *exist to bypass the infrastructural constraints of communication, so the problem is solved by using personal technological belongings instead of technology provided at work.*
***Photographs in EHR documentation*** *Low*	**Home care nurse colleagues** ***Referring to a photograph in a EHR message to a colleague*** *“[The home care nurse] writes a message [in the EHR system] to a colleague . . . regarding the redressing of a patient’s foot/heel . . . She asks what the colleague thinks about the healing process [of] the patient’s wound. She uploads a photo [of the wound from her smartphone by sending it to her work e-mail address and then] into the EHR documentation. [In the documentation] there are [earlier] photos [of the wound] as well, making it possible . . . to follow [the wound’s] healing process [over time] . . .”—Observation field note*	*The home care nurse experiences a* ***demand*** *for a second opinion. The nurse requests* ***instrumental support*** *from one of her colleagues, referring to the series of uploaded photographs. In doing so, the nurses gain better* ***control*** *over their mutual decision-making process.*

* The communication synchronicity of phone calls with GPs do not follow MST. The home care nurse-GP interaction is asynchronous, i.e. of low synchronicity. Message passing takes place rather than a direct phone call. The act of communication is pushed to a lower synchronicity level.

#### Oral Communication

Oral communication (see Table 2) is the verbal transmission of information through speech. This communication type consisted of *face-to-face meetings* and *phone calls*. These two media were the only ones identified that were used for synchronous communication. *Face-to-face meetings* involved two or more individuals and, as Table 2 shows, could be either prescheduled or spontaneous. Scheduled meetings were part of established work routines and involved actors situated both close and further away from the home care nurses organizationally. Spontaneous meetings often involved professional groups sharing the same physical workplace or common meeting grounds in the field. This was especially prevalent with nurse colleagues and homemaker service personnel. Between these actors, spontaneous synchronization about patients, as well as work in general, would happen upon sight. *Phone calls* were used in very much the same way, although they tended to be more purposeful and shorter in duration. Phone calls were often utilized for consultations and if tasks needed to be addressed right away.

As Table 2 shows, oral communication was important for home care nurses to manage work and keep their psychosocial work environment in balance. As Dennis et al. (2008) state, face-to-face meetings and phone calls, being more synchronous, are better suited to support the communication process of convergence than conveyance. If the home care nurses were free to choose, they would often prefer to communicate orally over the other two types of communication (written and visual). Thus, higher synchronicity was valued. This was not surprising since the continuous planning and adjustments of tasks were integral parts of the home care nurses day-to-day work. These activities relied on convergence to a large degree, since they required an intersubjective understanding. And yet, the nurses’ access to synchronous communication was very different within and outside the municipal organization.

Oral communication was overall a means for control and support for the home care nurses. The interactions were not free of demands. This was because it was necessary for the nurses to be available and to provide control and support in return as well. However, as shown in Table 2, the benefits of oral communication often outweighed its potential costs. In the municipal context, face-to-face meetings were more broadly deployed and more likely to occur. In one of the municipalities where different professional groups (nursing assistants, licensed practical nurses, coordinators, home care nurses, managers, occupational therapists, and physical therapists) were housed in the same building, they met more often, came by each other’s offices, and addressed problems synchronously. In general, home care nurses regularly synchronized about events that happened among themselves and with their closest collaborative partners, such as their colleagues and homemaker services. This gave the nurses a better chance to catch up, that is, to converge on events and proactively handle tasks. Phone calls were used as a long-distance complement to the face-to-face meetings as they also were used to facilitate convergence through synchronous communication.

Outside the municipal organization, though, there was less opportunities to arrange face-to-face meetings (especially with short notice). Here, phone calls became more important for convergence. General practitioners at community health centers would at best be available (scheduled) for face-to-face medical rounds once a week and then only for very limited periods of time (see Table 2).

For these short meetings, home care nurses needed to prepare and plan desired outcomes beforehand, strategizing on how to make sure that such an outcome occurred. Otherwise, they would have to make do with phone calls over which they passed messages through triage nurses or medical secretaries to get in contact with a physician. Communication with hospital professionals often took place over the phone in very much the same way as with those with community health centers. This behavior lowered the actual synchronicity of communication between the home care nurses and physicians in the regional health care system: from high/medium to low. This increased the demands on the home care nurses to manage communication, and simultaneously decreased their control over their work situation.

#### Written Communication

Written communication (Table 3) is the exchange of information through text or written language. Written communication had the most diverse types of media, including *handwritten notes*, *forms and physical documents, texting (SMS)*, *faxing, EHR documentation*, *EHR messaging*, *e-mail*, *digital medical signing*, and *communication portals*. Due to its low synchronicity, written communication was used to convey information.

*Handwritten notes* and *texting* served the same function of conveying short messages between professionals. Handwritten notes were on pieces of paper for varying purposes while texting was performed on smartphones. *Forms and printed documents* were physical pieces of paper used to convey information across contexts and professional groups. Both *faxing* and *communication portals* were used to convey confidential information about patients between home care and the regional health care system. Faxing was performed by sending and receiving physical documents scanned via fax machines or printers. By communication portals, we mean internet-based software systems used through web browsers to order different types of materials, medications or to convey information between health care professionals across the municipal regional boundary. The ones that were used consisted of a drug prescription service system, a national EHR system (in which home care nurses were only allowed to read, not write), and a system for creating coordinated care plans and care coordination regarding patient discharges from hospitals. *EHR documentation* and *EHR messaging* were carried out through the local system used in each municipality. *E-mails* went through an internationally well-known personal information manager software system. The EHR documentation was used for reading about reports on past events among home care nurses about actions taken since they last left their shifts. Access to the EHR documentation was limited to within the municipal context, and often also to one’s own profession. EHR messaging and e-mail correspondence were both used to convey information. However, conveying confidential information about patients was only allowed through EHR messaging, that is, within the municipal context. Communication portals, EHR documentation and messaging, and e-mail was performed on computers. *Digital medical signing* consisted of software used for delegation and signing of medications given to patients. Digital medical signing was performed on smartphones and administrated on computers.

Table 3 illustrates how home care nurses use written communication media. According to Dennis et al. (2008), written communication is better suited to support conveyance than convergence. This was further emphasized by the way the written media were used in practice. Written communication constituted a large part of the home care nurses’ administrative work tasks. In the mornings, nurses spent more time on written communication through reading. In the afternoons, more through writing.

On one hand, home care nurses had some ability to adapt their use of written communication to suit their own needs. This is exemplified by the two quotes about texting with homemaker services (see Table 3). Thus, if used in an effective and agreed upon manner, written communication would provide home care nurses with increased levels of control and support in their work. Other examples of this were when home care nurses read instructions from each other’s personal calendars or testimonies through the EHR documentation. This was a source of support and an opportunity to increase control over present and future work situations. By providing well written instructions, rich in content but short in length, home care nurses were better able to cover for each other and redistribute tasks among themselves if necessary.

On the other hand, restrictions existed in terms of what information was or was not allowed to be transmitted through each medium. This forced home care nurses to use certain kinds of media to convey certain information, especially confidential information regarding patients. Examples of such media were fax and communication portals. In these instances, even though a medium could not be used to a satisfactory degree (i.e., delayed answers), home care nurses were still forced to use them. This required the nurses to monitor responses and even remind the other party to answer. This, in turn, decreased their ability to obtain control over their work situation. This was evident in communication with the regional health care system, most especially with general practitioners at community health centers (see Table 3). To bypass this problem, in relation to the fax replies, the nurses would check for and distribute received faxes for both themselves and their colleagues whenever going to the fax.

#### Visual Communication

Visual communication (see Table 4) involves the use of images and other visual elements to transmit information. The nurses use of visual communication consisted of sending and uploading photographs via *picture messaging (MMS)* and *the local EHR system documentation* respectively. Like written communication, visual communication had low synchronicity, which meant that it was best suited to convey information. However, visual communication was never used alone but rather combined with oral or written communication to enhance the message. For example, picture messaging was used in combination with texting, phone calls, and face-to-face meetings. Uploaded photographs in the EHR documentation were used in combination with EHR messaging, EHR documentation, and face-to-face meetings. Picture messaging was performed on smartphones, while photographs in EHR documentation were accessed on computers.

As can be seen in Table 4, the main capability of visual communication was that it aided nurses and their collaborators in describing and tracking the status of wounds. To communicate visually appeared to be an important part in the chain of communication between homemaker services, home care, and community health centers. However, it was most common between the home care nurses and their homemaker service groups. Photographs were taken by either the home care nurses or the licensed practical nurses and nursing assistants in homemaker services. Picture messaging was enacted by home care nurses more often towards homemaker services and community health centers, while the use of uploaded photographs in EHR documentation was more exclusive among the home care nurses themselves.

Visual communication also aided home care nurses in their decision-making processes. Since the use of images supported both oral and written communication, it enriched the information they personally perceived they needed to convey. Images also supported convergence processes among the participants based on the much richer information that could be conveyed. The image thus became a form of “common ground” that created intersubjective understanding between the professionals (i.e., it created convergence). For example, the use of pictures made it easier to reach an agreement about the meaning of what was said or written about a wound. The risk of divergent interpretations and misunderstandings decreased. This was true among the home care nurses themselves, as well as in their work with other professional groups. The observed characteristics of media that supported visual communication lowered the demands placed on professionals to provide justifiable descriptions through speech or written communication alone, for example, of how a wound looked. Potential losses of information in transmission were thus averted, making the communication more effective. Used correctly, visual communication was found to be a source of support for home care nurses by increasing their control over their tasks.

A surprising observation was that unlike oral and written communication, visual communication was not a standardized work practice in any of the studied municipalities. This practice had been independently adopted across contexts by some home care nurses in each municipality. This indicated the potential usability of visual communication among home care nurses. However, visual communication was found to be poorly supported by the available technological infrastructure present for communication with the regional health care system. It could also be that the smartphones provided at work were not perceived as good enough to take high quality photographs. To avoid both shortcomings, home care nurses and other professionals across organizational boundaries used workarounds, for example, by picture messaging using their own private smartphones.

## Discussion

In this study, we have used MST to investigate how the type of communication media and its synchronicity affects home care nurses’ communication practice. Based on this analysis, we investigated the impact of the communication practice on the nurses’ psychosocial work environment from the perspective of the JDCS model. An interwoven discussion of the insights from the MST- and JDCS analysis is provided below.

A healthy work environment is associated with improved care quality and job satisfaction (Ma et al., 2015; White et al., 2020). Since communication is an integral part of nursing practice and a contributing factor to effective teamwork, the results from this study can provide a better understanding of how communication should be organized in order to provide effective care in a sustainable way.

The results pinpoint three main insights: 1) that the communication web have a protective function for the home care nurses, 2) that media and communication practice across organizational boundaries better suit conveyance than convergence, and 3) that control (in the JDCS sense) is gained by enabling or constraining communication through media. These insights are further discussed in the following sections.

### The Communication Web’s Protective Function for the Nurses

Bjornsdottir (2018) describes the idea of home care nurses creating and managing nets of services around each patient to ensure good quality care. The practice of home care nursing is also described as being collective where the boundaries of responsibility and expertise are fluid. This resonates well with the findings of our study where the home care nurses’ behavior of creating and managing the communication webs were at the very heart of their practice (Figure 2). By investigating the web’s functionality from a work environment perspective, this study extends Bjornsdottir (2018) results by finding evidence that the communication web potentially has a protective function for the nurses as well.

The conceptualization of home care nursing as collective work pinpoints the inherent interdependency of work and professionals in this field. This conceptualization showcases the benefits of supporting each other to support oneself (e.g., see Table 2). This resonates well with the results from other studies where nurses find support through well-functioning teamwork in intra- and interprofessional constellations (e.g., San Martín-Rodríguez et al., 2005; Shaw et al., 2016; Tourangeau et al., 2014). Our results indicate that the home care nurses were supportive of one another to safeguard the quality of care provided for their patients. They did this by working proactively to prevent problems from occurring or escalating. Early engagement in communication and collaborative deliberations was highly preferable since catching problems at early stages often required fewer resources. Additionally, this benefited work efficiency.

However, the communication web’s supportive function required work to be maintained. Previous studies show that nurses play an important role in teamwork by holding patient care together (Lee et al., 2019; Sun et al., 2019; Trude Fløystad et al., 2018). This was also the case in our results (see Tables 2 and 3). In a sense, home care nurses took on the role as the driving and coordinating forces within the communication web. From one perspective this is something home care nurses find attractive in their profession (De Groot et al., 2018). But as our results show, it also introduced pressure and demands on the nurses to take on the role of representing the patients’ interests and needs in their interactions with the regional health care system. For the sake of their patients, the home care nurses had to be the driving force of communication to manage the shortcomings of the divided responsibility for care in the Swedish health care system.

### Media and Communication Practices Across Organizational Boundaries Better Suit Conveyance Than Convergence

From home care nurses’ perspectives, communication across organizational boundaries, such as with community health centers, was often arranged to support conveyance. Yet, the results showed that communication with high synchronicity, that is, that supported convergence, was preferred by the home care nurses as it provided a means to reduce uncertainty and achieve control over their work, thus reducing stress (Karasek, 1979; Karasek & Theorell, 1990). This also resembles the findings of Bjornsdottir (2018), where formal and informal conversations are described as important for practice.

Synchronous communication best supported convergence and convergence is considered important for creating shared mental models among group members, and for creating shared understandings of goals, tasks and specific roles in relation to collective tasks (Mathieu et al., 2000; McComb & Simpson, 2014). Convergence was important in our study for the sake of the work environment and the needs that professionals experience in home care; it assisted them in reaching understandings of patient care tasks as well as the needs of one another as professional groups.

The results show that home care nurses use a variety of media for communication (see Table 1 and Figure 2). This is not necessarily a bad thing in itself. Dennis et al. (2008) presents the idea that the “best medium” for a given situation may in fact be a combination of media. Combined media can complement or even enhance communication if used correctly. For example, oral media can be combined with written and/or visual media to provide complementary information and to achieve a deeper understanding of what needs to be done. An example of this was the convergence obtained by applying visual communication to better understand the status of wounds (see Table 4). The independent application of visual communication in different municipalities indicated that the nurses found it useful. It enabled them to not only share information of an event but to “experience” it. In one sense, what a picture conveys of a wound was much like the experience of seeing the wound in real life.

Apart from face-to-face meetings, handwritten notes, and physical documents, communication for nurses was found to be highly technologically mediated (see Table 1). This showed how strong the dependency on technology is, and how communication through technology plays an important role in modern Swedish home care work. The utilization of technology-mediated communication indeed enables interactions between professional groups that are normally separated by geographic, time, and organizational boundaries. This enables health care to move outside of traditional institutions and into patient home environments. From a work environment perspective, though, this does not mean that technology-mediated communication is beneficial on its own for the professionals using them. Apart from phone calls, the technological media used by the home care nurses in this study best provided conditions for communication of low synchronicity and conveyance (see Table 1 and Figure 2).

The home care nurses had better means for higher synchronicity in communication with other professionals on the municipal level (see Figure 2). This was due to the lower thresholds to engage in oral communication. Outside the municipality, especially with the regional health care system, communication became more asynchronous. Written communication was the default communication type in this organizational interface. The systematic practice of gatekeeping and message passing here was problematic for the home care nurses (see Tables 2 and 3). For example, communication with community health centers took the form of message passing even when contact was made over the phone. Communication was thus forced to become low in synchronicity even when the medium enabled a higher level of synchronicity.

### Control Is Gained by Enabling or Constraining Communication Through Media

Home care nurses value control (Larsson et al., 2022). Previous research has expressed this as an emphasis on autonomy, self-direction, flexibility, and authority (Bjornsdottir, 2018; De Groot et al., 2018; Larsen et al., 2017; Lindblad et al., 2018; Maurits et al., 2017; Tourangeau et al., 2014). As previously stated, control over the work situation was also associated with less stress (Karasek, 1979; Karasek & Theorell, 1990). The home care nurses had some degree of autonomy by which they could tailor their communication practice. Some nurses utilized texting to enable communication with homemaker services (see Table 3). They experienced the option of texting as being advantageous by providing homemaker services the possibility to send short summary notes if necessary. The nurses could later check these notes when convenient. Other nurses chose to actively avoid texting altogether because it could pose the risk of forgetting the message since no reminders were given (see Table 3). By limiting communication to specific media, home care nurses gained a better ability to foresee where to expect communication about certain topics. In other words, the nurses exercised power through their hierarchical status (Thunborg, 1999). Consequently, they gained control and reduced demands for themselves. They took actions that could reduce their own stress levels at the cost of others.

However, if a physician would call, home care nurses would answer the phone even if busy. In a similar manner to the home care nurses, though, through gatekeeping via triage nurses and medical secretaries, the general practitioners and other physicians distanced themselves. By putting up gatekeepers towards the home care nurses, the day-to-day communication over the municipal-regional interface was pushed to be asynchronous and almost exclusively enacted through message passing via faxing and phone calls (see Tables 2 and 3). This is a challenge for home care nurses from a work environment perspective due of the hierarchal dependency they have on physicians, especially the general practitioners at community health centers with whom they interact the most (Thunborg, 1999). In this case, the home care nurses were the ones paying the price of lost control and increased demands due to limitations in communication, which could increase their stress levels (Karasek & Theorell, 1990).

In terms of the JDCS model (Karasek & Theorell, 1990), more predictable communication can increase levels of control while decreasing demands to monitor media in parallel. Controlling the possibilities for others to initiate communication may be a way to guard a worker’s time and resources and thus reduce the complexity of work. Distancing yourself from others who depend on you make your own work less demanding and more predictable because you have taken control over the communication. In turn, though, you may cause problems for them. For example, general practitioners at community health centers probably saved time by limiting the home care nurses’ communication initiatives, but they also created significant loss of time and control for the nurses. This strategy to gain control through communication constraints is a type of local optimization, that is, tailoring a practice according to the conditions in a specific work context, but not to the conditions in work as a whole. These constraining activities seemed to favor conveyance (Dennis et al., 2008). Convergence, which is fundamental to establish common ground and intersubjectivity between people, could be harder to achieve across the municipal-regional boundary (Dennis et al., 2008; Gillespie & Cornish, 2010). Thus, constraining communication to gain control may be a beneficial strategy locally in the short-term if work conditions are strained. But when used as a long-term strategy, it will be a potential source of increased stress for professionals’ having a status of dependency in their profession. This may rather trigger conflicting interests that jeopardize possibilities for reaching well-functioning collaboration and teamwork across organizational boundaries.

Communication is important for teamwork and collaboration in health care (Härgestam et al., 2013; Hewitt et al., 2015; Larsson et al., 2022; Lingard et al., 2006; Rydenfält et al., 2017; Suter et al., 2009). This study indicates that the choice of communication media and organization of communication can affect the psychosocial work environment of the nurses. Thus, the results from this study can inform the design of communication practice in home care nursing and contribute, both to improved care quality, and a better work environment for home care nurses.

## Limitations

This study was conducted from the perspective of municipal home care nurses. However, the other identified professional groups had communication pathways of their own. Thus, it would be of considerable interest to conduct the same type of study on them as well. Due to its qualitative and ethnographic nature, this study only described the situation in four of Sweden’s 290 municipalities. However, the method resulted in an in-depth analysis of the situation in these four municipalities. Thus, a deep understanding was achieved of the home care nurses’ situation in these municipalities. There may be deviations in detail across Swedish municipalities, but this study nevertheless helps depict the overall working conditions and communication practices of municipal home care nurses in Sweden. The focus has been on how synchronicity of communication through individual media can influence the psychosocial work environment of home care nurses, not the synchronicity of their tasks as a whole. The completion of tasks may include the use of a combination of media, not just one, and be spread out over time. The resulting synchronicity of task completion among professional groups and its effects on their psychosocial work environment would be of considerable interest to study in the future.

## Conclusions

Home care nurses are at the heart of communication in the home care context, tying together the communication web between professional groups. Results indicate that the web can have a protective function for the nurses by providing control and support. This function is conditional, though, and work is needed to maintain it.

Home care nurses use a multitude of media for communication. Results show synchronous communication to be highly valued and important to cope with work. Nevertheless, asynchronous communication is enforced when reaching outside the municipality, for example, towards the regional health care system.

A lack of fit between communication and task can lead to increased demands and loss of control for nurses at work. However, media that enable the right communication processes (conveyance or convergence) in relation to the tasks can contribute positively to the nurses’ psychosocial work environment. The ideal is to match requirements for communication processes among professional groups with media to lower demands and increase control and support at work. This is to promote a healthy work environment.

When influence over media choice is used to gain one-sided control, it can negatively affect others in a dependent situation. This is problematic for professions like nurses that depend on physicians. Communication and work conditions in the home care context should be designed with a holistic perspective in mind, to support the home care nurses’ position, and promote well-functioning interprofessional teamwork across organizational and professional boundaries. This is crucial to meet the rising demands for quality health care in home environments, as well as to establish a healthy work environment for home care nurses.
